# Effectiveness of health education intervention in improving knowledge, attitude, and practices regarding Tuberculosis among HIV patients in General Hospital Minna, Nigeria – A randomized control trial

**DOI:** 10.1371/journal.pone.0192276

**Published:** 2018-02-22

**Authors:** Chindo Ibrahim Bisallah, Lekhraj Rampal, Munn-Sann Lye, Sherina Mohd Sidik, Normala Ibrahim, Zubairu Iliyasu, Michael Ochigbo Onyilo

**Affiliations:** 1 Department of Community Health, Faculty of Medicine and Health Sciences, Universiti Putra Malaysia, Serdang, Selangor, Malaysia; 2 Department of Public Health, Ministry of Health, Minna, Niger state, Nigeria; 3 Department of Psychiatry, Faculty of Medicine and Health Sciences, Universiti Putra Malaysia, Serdang, Selangor, Malaysia; 4 Department of Community Medicine & Centre for Infectious Diseases Research, Bayero University Kano, Nigeria; 5 Niger state Agency for the control of AIDS, Minna, Niger state, Nigeria; Centro de Investigação em Saúde de Manhiça, MOZAMBIQUE

## Abstract

**Introduction:**

The risk of development of active TB in HIV-infected individuals is 20–37 times higher than those that are HIV negative. Poor knowledge of TB amongst people living with HIV has been associated with high transmission.

**Objectives:**

To determine the effectiveness of a new health education intervention module in improving knowledge, attitude, and practice (KAP) regarding tuberculosis among HIV patients in General Hospital Minna, Nigeria.

**Methods:**

A randomized control trial was carried out from July 2015 to June 2017. A random number generating program was used to allocate 226 respondents into 2 groups. The intervention group received health education regarding tuberculosis using the developed module. The control group received the normal services provided for HIV patients. Data were collected from December 2015 to September 2016 at baseline, immediate post intervention, three, six and nine months. The outcome measures were knowledge, attitude, and practice.

**Results:**

There was no significant difference with respect to socio-demographic characteristics, KAP of the respondents in the intervention and control group at baseline. However, there was significant improvement in knowledge in the intervention group compared to the control group, group main effect (F = (1,218) = 665.889, p = 0.001, partial ἠ^2^ = 0.753, d = 5.4); time (F = (3.605, 218) = 52.046, p = 0.001, partial ἠ^2^ = 0.193, d = 1.52) and interaction between group with time (F = (3.605, 218) = 34.028, p = 0.001, partial ἠ^2^ = 0.135, d = 1.23). Likewise, there was significant improvement in attitude, group main effect (p = 0.001, d = 1.26) and time (p = 0.001, p, d = 0.65). Similarly, there was improvement in practice, group main effect, time, and interaction of group with time (p < 0.05).

**Conclusion:**

The health education intervention program was effective in improving KAP regarding tuberculosis among HIV patients.

## Introduction

Tuberculosis (TB) is the commonest cause of death in acquired immune deficiency syndrome (AIDS) patients [1]. The rising incidence of TB among human immunodeficiency virus (HIV) patients in Nigeria poses a great threat to TB control. The risk of development of active TB in HIV-infected individuals is up to 20–37 times higher than those that are HIV negative [2, 3]. In 2015, out of 87,211 TB cases in Nigeria, 14,846 HIV-positive TB patients were registered for treatment and care [4]. Poor knowledge of TB amongst people living with HIV is associated with high transmission and delay in health-seeking behavior [5]. The effect of health education intervention program has not been evaluated in Nigeria. There is no structured health education intervention program on behavior modification that is directed and specific for HIV patients regarding tuberculosis in Nigeria. Advocacy, Communication, and Social Mobilization (ACSM), a component of TB control program in Nigeria was evaluated in 2012 and found to be largely ineffective [6]. The objective of this study was to develop and implement a new health education intervention manual and evaluate its effectiveness in improving knowledge, attitude and practices regarding tuberculosis among HIV patients enrolled for treatment in General Hospital Minna, Nigeria.

## Methods

### Study design and location

A Single-blind, parallel group, randomized control trial was conducted between July 2015 to June 2017. Data were collected from 4^th^ December 2015 to 3^rd^ September 2016 at baseline, immediate post intervention, three, six and nine months at General Hospital, Minna, Niger state, North-Central, Nigeria.

### Sample size calculation

We estimated the sample using the formula for calculating sample size in hypothesis testing by comparing two means as described by Lemeshow *et al* 1990 [7]. This was done for the three outcome variables of this study, of which knowledge provided the highest sample size and hence was adopted for the study. We estimated that to detect a difference of 15% in mean knowledge scores between the 2 groups from the baseline value of 4.9 [8] over a 9 months period, a minimum of 186 respondents would provide 80% power assuming a type 1 error rate of 5%. We adjusted for 18% attrition rate and arrived at a total sample size of 226 for the study.

### Participant recruitment and eligibility

A simple random sampling method was used to select eligible participants. The sample frame comprised of a list of 603 HIV patients receiving treatment and care in the preceding two years (from 1st July 2013 to 30th June 2015) at the study center. Twenty-one patients were dropped for not meeting the eligibility criteria. The random sample was selected using a computer-based random number generator (http://www.randomizer.org) used by earlier researchers [9]. A total of 226 participants were randomly selected while ensuring an equal chance for each potential study participant using same computer generator program. The inclusion criteria were registered HIV patients, including AIDS patients accessing treatment and care at Minna, General Hospital, age 18 years and older. HIV patients co-infected with tuberculosis or psychiatric disorders or cognitively impaired persons were excluded.

### Randomization and blinding

**Fig 1:**
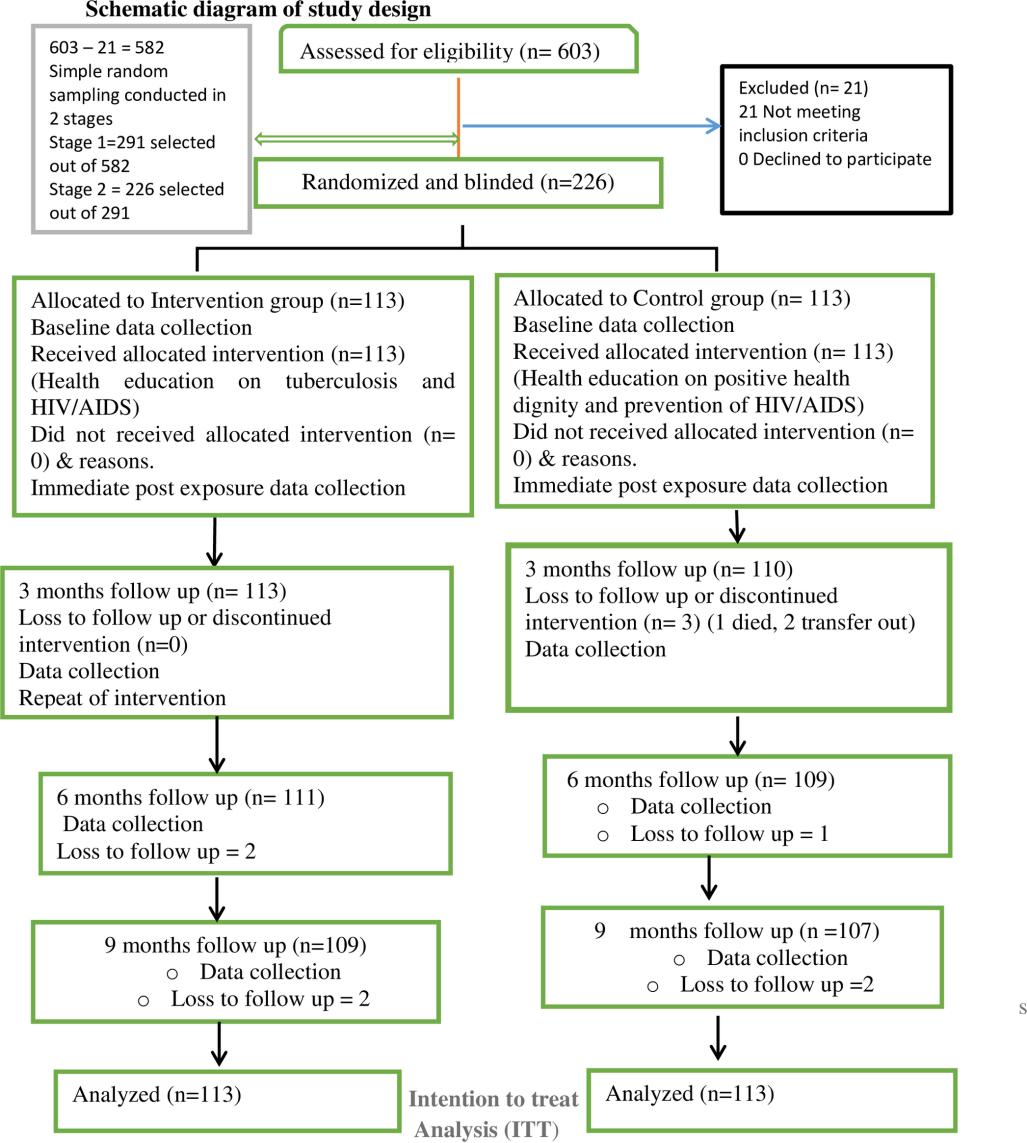
Flow chart of respondents in a randomized control trial conducted among HIV patients in General Hospital, Minna, Nigeria.

An independent statistician generated the random allocation sequence using random number generator program (http://www.randomizer.org) to allocate the sampled population into an intervention and a control group using simple randomization technique with a ratio of 1:1, each group having 113 participants. The written allocation was used with identification codes in sealed brown opaque envelopes. The code numbers were used to identify participants on the questionnaire while maintaining confidentiality. The investigator enrolled and assigned participants to the two groups. The participants were blinded. They were not aware of the random allocation or the hypothesis that was tested. Fig 1 shows the flow chart of the randomization and blinding.

### Intervention module

The health education intervention module on knowledge, attitude, and practice regarding tuberculosis among HIV patients was developed through a process of consultations with a group of experts in preventive medicine and behavior modification. The module was developed based on the Information, Motivation, and Behaviour skills model [10]. The information component of the module provided information on basic facts about tuberculosis, modes of transmission, prevention, risk factors, vulnerability and misconception related to TB. The motivation component of the model was aimed at countering misconceptions about tuberculosis leading to positive attitudinal change. The preceding steps then motivate individuals to access screening and preventive services provided free of charge to HIV patients. Knowledge acquired during the training reinforces preventive behavior skills.

### Facilitators training

The training was attended by six facilitators who met prior selection criteria. The criteria included at least one-year experience working in HIV/AIDS-TB and previous training conducted in these areas. The training involved lectures using PowerPoint slides, brainstorming, discussions, question and answer sessions with feedback from the facilitators. A refresher training was held a day before the intervention. Each facilitator was given the training manual and PowerPoint presentations as a guide. The data collection tool used for the study was also presented and explained to ensure quality control during data collection.

### Intervention group

Health education intervention program on TB module was delivered at General Hospital, Minna to recruited HIV patients on the 4^th^ December 2015. The method and materials that were used to deliver the intervention included lectures using PowerPoint slides and posters. There were sessions for discussions, questions and answers to re-enforce learning. The group had 113 participants which were further subdivided into a class of not more than thirty-eight participants for ease of delivery. A total of seven facilitators were involved in the intervention process including the researcher. A total of one hundred and thirteen participants in the intervention (treatment) arm received the module which lasted for a period of six hours. A booster session was delivered at three months.

### Control group

The control group also had 113 participants. The group received normal health education services provided by the Ministry of Health, positive health, dignity and prevention regarding HIV/AIDS on the second day lasting six hours. The same facilitators were involved in the delivery of the program for the control group using same methods as in the intervention group except the manual used was on Positive health, dignity, and prevention regarding HIV/AIDS.

### Data collection

Data collection was carried out from December 2015 to September 2016. A validated, pretested and self-administered questionnaire consisting of six sections was used. The questionnaire was a modified version of WHO tool: a guide to developing knowledge, attitude and practice surveys [11]. The sections A, B, C, D, E, and F covered socio-demographic variables, TB-related knowledge, attitude towards TB, practices related to TB, anxiety, and depression, clinical and laboratory parameters respectively. The section of the questionnaire on anxiety and depression was adopted from the study on the validation of Hospital anxiety and depression rating scale among HIV/AIDS patients at Kano, Nigeria [12]. Section A had 13 statements on socio-demographic variables. The section on TB knowledge had 24 statements with ‘Yes' or 'No' options. Section C had 9 questions relating to the attitude of participants towards tuberculosis. The responses were on a 5-point Likert scale with the options: strongly agree, agree, neutral, disagree and strongly disagree. Section D had statements on the practice of patients relating to tuberculosis prevention with 'Yes' or 'No' options. Section E had statements on the hospital anxiety and depression (HAD) scale with the options 0,1,2,3 for each statement. The last section E contained information relating to the patient clinical and laboratory parameters. Results of the reliability test carried out showed Cronbach's coefficient alpha for knowledge, attitude, practice, anxiety, and depression was 0.847, 0.777, 0.792, 0.733 and 0.811 respectively. Data were collected from respondents by the investigator at baseline, immediate post intervention, three, six and nine months' post-intervention. Those that could not read or write were assisted and questions were read out to them in the local language (Hausa).

### Measurement of outcome variables

The outcome variables for this study were knowledge, attitude, and practice regarding tuberculosis. Knowledge had 24 questions and had 'Yes' or 'No' options. Correct answers attracted one mark while wrong answers were scored zero. A respondent could get scores within the range of 0–24 scores. Attitude had 9 questions measured on a five-point Likert scale with strongly disagree = 1, Disagree = 2, Neutral = 3, Agree = 4, and strongly agree = 5. The scores ranged from 9 to 45. Practices had 9 questions with 'Yes' or 'No' options. Correct responses had one point while zero was awarded for wrong answers. The minimum was 0 and the maximum score was 9. The data were assessed to determine the extent of missing outcome data. In the three outcomes variables, there were a total of 47,460 responses, with each respondent having 210 responses. A total of 576 responses were left blank from baseline to the end of the research representing 1.2% of the total data with no consistencies in missing responses observed.

### Data analysis

Data analysis was carried out using Statistical package for social sciences (SPSS) version 22 (IBM 2014). Parametric test such as Independent t-test, mixed design ANOVA and Nonparametric tests (Chi-square and Fisher exact test) were used to analyse the data. The level of significance of 5% was used. Chi-square and Fisher exact test were used to analyse categorical variables. Sensitivity analysis was done to determine violations of the missing at random assumption. Mixed design ANOVA was the major analytical method used to determine the effectiveness of the intervention [13]. Analysis of outcome variables was done by intention to treat (ITT) analysis. Partial eta squared (ἠ^2^) and Cohen d were the measures of effect size. The strength of partial eta square (ἠ^2^) was interpreted as small effect = 0.01, moderate effect = 0.06, larger effect = 0.14 and Cohen d as small effect (d = 0.2), medium (d = 0.5), and large (d = 0.8) [14, 15].

### Ethical issues

Ethical clearance to conduct the study was obtained from Universiti Putra Malaysia, Ethics Committee for Research Involving Human Subjects and the Niger State Ministry of Health human research ethics committees. Informed written consent was obtained from each participant. In addition, the trial was registered with Pan African Clinical trial registry with registration number PACTR201603001403923.

## Results

### Response rate

The response rate at the end of 9 months intervention was 95.5%. Two hundred and twenty-six HIV-positive patients participated in the study. Ten patients were lost to follow-up.

### Treatment of missing data

Missing data from dropouts or loss to follow-up were replaced with imputation using the mean substitution method in line with the principle of intention to treat analysis [16]. However, to evaluate the robustness of our results and the conclusion of our intervention, a sensitivity analysis was conducted. This was done to determine the consistency of results of sensitivity analysis with the chosen method of handling of the missing data [17]. The results of sensitivity analysis are summarized in Table 1.

**Table 1:** Summary of mixed design ANOVA for tuberculosis knowledge, attitude and practice scores (between and within-subject effects) (N = 216).

Outcome measure	Source of variance	Type 111 Sum of squares	Degree of freedom	Mean square	F	p-value	partial ἠ^2^	Cohen d
**Knowledge**	Group	9071.398	1	9071.398	660.202	0.001	0.760	5.31
	Time	2369.075	3.667	644.383	52.387	0.001	0.201	1.5
	Group x time	1665.45	3.667	437.039	35.530	0.001	0.146	1.24
**Attitude**	Group	1000.046	1	1000.046	31.314	0.001	0.131	1.16
	Time	803.978	3.587	224.153	9.996	0.001	0.046	0.66
	Group x time	167.790	3.587	46.781	2.086	0.089	0.010	0.30
**Practice**	Group	370.990	1	370.990	139.778	0.001	0.402	2.45
	Time	215.276	3.632	59.273	21.881	0.001	0.095	0.97
	Group x time	124.225	3.632	34.204	12.627	0.001	0.057	0.73

Significant at p<0.05; Partial ἠ^2^—partial eta square; P-values were calculated using mixed design ANOVA; Group x time interaction represents the treatment effect as the difference in change-from-baseline between the two groups; F-statistics

### Socio-demographic characteristics by groups

Tables 2 and 3 shows the socio-demographic characteristics and psychosocial factors (Anxiety and Depression) of the study respondents in the intervention and control groups at baseline. The results showed that there was no significant difference between the two groups. The two groups were comparable at baseline.

**Table 2:** Socio-demographic characteristics of the respondents.

Characteristics	Total N = 226	Intervention n = 113	Control n = 113	Test type	p-value
**Age**					
Mean, SD	37.98 ± 10.43	37.92 ± 10.84	38.04 ± 10.04	t-test	0.93
95% CI	36.6–39.34	35.89–39.9	36.10–39.92		
**Gender**					
Male	94(32.7)	32(28.3)	42(37.2)	χ^2^	0.16
female	152(67.3)	81(71.7)	71(62.8)		
**Ethnicity**					
Hausa	45(19.9)	26(23.0)	19(16.8)	χ^2^	0.51
Nupe	44(19.5)	20(17.7)	24(21.2)		
Gwari	46(20.4)	20(17.7)	26(23.0)		
others	91(40.3)	47(41.6)	44(38.9)		
**Educational level**					
No formal education	48(22.2)	24(21.2)	24(21.2)	χ^2^	0.70
Primary	51(22.6)	29(25.7)	22(19.5)		
Secondary	84(37.2)	39(34.5)	45(39.8)		
College/University	43(19.0)	21(18.6)	22(19.5)		
**Religion**					
Islam	135(59.7)	70(61.9)	65(57.5)	χ^2^	0.50
Christianity	91(40.3)	43(38.1)	48(42.5)		
**Occupation**					
Civil servant	39(17.3)	14(12.4)	25(22.1)	Fisher’s exact test	0.072
Farmer	14(6.2)	4(3.5)	10(8.8)		
Housewife	55(24.3)	32(28.3)	23(20.4)		
Students	19(8.4)	8(7.1)	11(9.7)		
Trader	71(31.4)	40(35.4)	31(27.4)		
Not employed	23(10.2)	14(12.4)	9(8.0)		
others	5(2.2)	1(0.9)	4(3.5)		
**Marital status**					
Single	30(13.3)	15(13.3)	15(13.3)	Fisher’s exact test	0.49
Married	162(71.7)	77(68.1)	85(75.2)		
Separated	8(3.5)	6(5.3)	2(1.8)		
Divorced	4(1.8)	3(2.7)	1(0.9)		
widowed	22(9.7)	12(10.6)	10(8.8)		
**Place of residence**					
Urban	144(63.7)	69(61.1)	75(66.4)	χ^2^	0.41
rural	82(36.3)	44(38.9)	38(33.6)		

Significant at p <0.05; SD- Standard Deviation; χ^2^- Chi Square, CI- Confidence interval

**Table 3:** Baseline comparison of psychosocial factors (anxiety and depression) of the study respondents at baseline.

Characteristics	Total N = 226	Intervention n = 113	Control n = 113	Test type	p-value
**Anxiety**					
Mean, SD	10.07 ± 3.50	9.65 ± 3.62	10.48 ± 3.35	t-test	0.075
95% CI	9.61–10.53	8.96–10.33	9.86–11.11		
**Depression scores**					
Mean, SD	9.40 ± 2.42	9.59 ± 2.42	9.22 ± 2.42	t-test	0.250
95%CI	9.08–9.72	9.14–10.04	8.76–9.67		

Significant at p < 0.05; SD- Standard Deviation; CI—confidence interval

### Mean knowledge scores

Exploratory data analysis performed on knowledge, attitude and practice scores showed that data was normally distributed among the respondents. At baseline, there was no significant difference in mean knowledge scores between the intervention and the control groups (mean = 15.26 ± 4.65 vs. 14.35 ± 4.32, p = 0.13). Thereafter, there was significant increase in mean knowledge scores in intervention group compared to control group immediate post-intervention, three, six and nine months (21.92 ± 2.06 vs. 13.32 ± 2.92, p< 0.001, 21.82 ± 2.56 vs. 15.34 ± 3.60, p < 0.001, 22.95 ± 1.47 vs. 15.86 ± 4.84 p <0.001 and 22.70 ± 1.98 vs. 15.19 ± 4.13, p<0.001) respectively.

### Mean attitude scores

The results showed that there was no significant difference in mean attitude scores between the intervention and the control groups (29.64 ± 5.71 vs. 29.08 ± 4.80, p = 0.43) at baseline. However, there was a significant higher mean attitude scores in the intervention group compared to the control group immediate post-intervention, three, six and nine months (33.14 ± 3.76 vs. 29.52 ± 4.67, p < 0.001, 33.12 ± 4.50 vs. 30.67 ± 5.34, p < 0.001, 32.86 ± 4.45 vs. 30.84 ± 4.97, p = 0.001) and (33.07 ± 4.06 vs.30.57 ± 4.47, p = 0.001) respectively.

### Mean practice scores

The results also showed that there was no significant difference in the mean practice scores between the intervention and the control groups at baseline (6.01 ± 1.91 vs. 6.00 ± 1.90, p = 0.945). There was a significant increase in mean practice scores in the intervention group compared to the control immediate post-intervention, three, six and nine months (7.87 ± 2.32 vs. 5.89 ± 1.62, p < 0.001, 7.79 ± 0.93 vs. 6.36 ± 1.64 p < 0.001, 8.22 ± 0.90 vs. 6.79 ± 1.40, p = 0.001 and 8.08 ± 1.13 vs. 6.69 ± 1.27, p-value 0.001) respectively (Table 4).

**Table 4:** Main effect of intervention on mean knowledge, attitude and practice scores regarding tuberculosis at baseline to follow-up.

Outcome measure	Time	Intervention group	Control group	t(224)	p-value
		mean ± SD	mean ± SD		
**Knowledge**	Baseline	15.26 ± 4.65	14.35 ± 4.31	1.527	0.13
	Immediate post intervention	21.92 ± 2.06	13.32 ± 2.92	25.530	0.001
	3 months	21.82 ± 2.56	15.34 ± 3.60	15.565	0.001
	6 months	22.95 ± 1.47	15.86 ± 4.84	14.879	0.001
	9 months	22.70 ± 2.10	15.19± 4.13	17.559	0.001
**Attitude**	Baseline	29.64 ± 5.71	29.08 ± 4.82	0.793	0.43
	Immediate post intervention	33.14 ± 3.76	29.52 ± 4.67	6.413	0.001
	3 months	33.12 ± 4.50	30.67 ± 5.34	3.725	0.001
	6 months	32.86 ± 4.45	30 84 ± 4.97	3.206	0.001
	9 months	33.07 ± 4.06	30.57 ± 4.72	4.397	0.001
**Practice**	Baseline	6.01 ± 1.91	6.00 ± 1.90	0.070	0.95
	Immediate post intervention	7.87 ± 2.32	5.89 ± 1.62	7.429	0.001
	3 months	7.79 ± 0.93	6.36 ± 1.64	8.024	0.001
	6 months	8.22 ± 0.90	6.79 ± 1.40	9.073	0.001
	9 months	8.08 ± 1.13	6.69 ± 1.27	8.625	0.001

SD, standard deviation. Significant at p value < 0.05. P-value obtained by student t-test for independent samples.

### Magnitude of effects of intervention on knowledge, attitude, and practice

Results showed significant main effect for group (p = <0.001, partial ἠ^2^ = 0.753, Cohen d = 5.4), time (p <0.001, partial ἠ^2^ = 0.193, d = 1.52) and the interaction of group with time (p <0.001, partial ἠ^2^ = 0.135, d = 1.23) for tuberculosis related knowledge. The findings of analysis on mean attitude scores showed significant main effect for group (p < 0.001, ἠ^2^ = 0.141, d = 1.26) and time (p <0.001, partial ἠ^2^ = 0.043, d = 0.65). No significant interaction effect of group with time (p = 0.077, partial ἠ^2^ = 0.010, d = 0.31). Similarly, results revealed significant main effect for group (p < 0.001, ἠ^2^ = 0.392, d = 2.56), time (p <0.001, partial ἠ^2^ = 0.10, d = 1.02) and interaction effect of group with time (p = 0.001, partialἠ^2^ = 0.053, d = 0.74) for tuberculosis related practices (Table 5).

**Table 5:** Summary of mixed design ANOVA for tuberculosis knowledge, attitude and practice scores (between and within subject effects) (N = 226).

Outcome measure	Source of variance	Type 111 Sum of squares	Degree of freedom	Mean square	F	p-value	Partial ἠ^2^	Cohen d
**Knowledge**	Group	9182.618	1	9182.618	662.889	0.001	0.753	5.4
	Time	2389.10	3.605	662.637	52.046	0.001	0.193	1.52
	group x time	1561.998	3.605	433.234	34.028	0.001	0.135	1.23
**Attitude**	Group	1156.366	1	1156.366	35.921	0.001	0.141	1.26
	Time	776.448	3.547	218.878	9.735	0.001	0.043	0.65
	group x time	174.108	3.547	15.547	2.183	0.077	0.010	0.31
**Practice**	Group	377.483	1	377.483	140.273	0.001	0.392	2.56
	Time	234.684	3.632	64.616	24.201	0.001	0.100	1.02
	group x time	118.258	3.632	32.560	12.195	0.001	0.053	0.74

Significant at p<0.05. Partial ἠ^2^, partial eta square. Group x time interaction represents the treatment effect as the difference in change-from-baseline between the two groups. P-values were calculated using mixed design ANOVA. F-statistics.

## Discussion

The present study utilized information, motivation and behavior based intervention program and treatment fidelity as strategies to improve knowledge, attitude and practice regarding TB among HIV patients in the intervention group. The results of this study provide evidence that significant improvement in knowledge, attitude, and practice regarding TB among HIV patients can be achieved through a structured health education intervention. We believe that the observed changes in the outcomes can be attributed to the effect of the intervention delivered. However, studies that evaluated the effect of health education intervention on knowledge, attitude and practice regarding TB among HIV patients are scarce. We were therefore constrained in comparing the effect sizes of our intervention with other studies among HIV patients. Further research is required to evaluate the effect of health education program regarding TB especially in countries with high HIV prevalence with increasing burden of TB among HIV patients.

### Effectiveness of intervention on knowledge

This study and other studies have consistently shown that knowledge of HIV patients regarding tuberculosis was poor and recommended a specific and targeted education among this at-risk group [5, 18, and 19]. In this research, significantly higher mean knowledge scores seen in the intervention group compared to the control group revealed that there was an improvement in knowledge among respondents that received the intervention. The effect size for this study based on the group x time interaction (d = 1.23) was found to exceed Cohen’s (1988) convention for a large effect (d ≥ 0.80) [15]. However, there was no significant difference in the effect of the intervention on sex and age. This study is in agreement with the report of a study conducted in Egypt among a sample of the general population which showed significant improvement in knowledge regarding tuberculosis [8]. Similarly, our intervention recorded significant improvement in knowledge comparable to the report of studies on the impact of simple and peer-led versus teacher-led education intervention on knowledge of respondents regarding tuberculosis [20, 21]. Consequently, effort in providing effective health education intervention to this vulnerable group regarding this disease will have a positive impact.

### Effectiveness of intervention on attitude

The attitude of respondents regarding tuberculosis improved in the intervention group compared to the control group with small effect size. However, the effect of health education on attitude was not influenced by sex or age of the respondents. The effect size for this study based on the group x time interaction (d = 0.31) was found to be within Cohen’s (1988) convention for a small effect (d = 0.20) [15]. Our intervention had effect on the improvement of the attitude of respondents toward TB similar to the report of an intervention among students of secondary school [21]. Attempts at improving attitude toward tuberculosis will eventually have a positive impact on tuberculosis prevention and control among HIV patients.

### Effectiveness of intervention on practice

Health education intervention was significantly effective in improving preventive practices of respondents that had the intervention compared to the control group. The effect size for this study based on the group x time interaction (d = 0.74) was found to be within Cohen’s (1988) convention for a medium effect (d = 0.5) [15]. This study and other studies have shown that preventive practices regarding tuberculosis are still suboptimal despite a high level of awareness and availability of preventive services [22–24]. The finding of this research is consistent with the report of other studies that have demonstrated the effectiveness of health education intervention in improving preventive practices regarding tuberculosis [21, 25]. In consideration of the importance of preventive practice in tuberculosis prevention and control as a requirement, the need for information and positive attitudinal change towards the disease is paramount. Further studies are required to determine the effect of sex and age on health education intervention on knowledge, attitude and practice regarding tuberculosis among HIV patients.

### Treatment fidelity and compliance

The intervention was delivered by six trained health professionals and supervised by the research team to ensure compliance with the study protocol in its delivery. The intervention lasted for six hours in each of the group and a booster session was delivered at three months. The follow up was done regularly at intervals of three months apart to the end of the intervention that lasted for nine months. Despite, conducting the training in two separate days for the intervention and control group and blinding of the respondents to group allocation, there was evidence of some level of contamination as indicated in improvement in mean knowledge and practice scores in the control group. Similar observation of possible contamination of the control group has also been reported in another intervention study among HIV patients [26]. Respondents in our study, in the intervention and control groups were drawn from the same study base where they attend the clinic for treatment and care with high possibility of interaction during the study period. This could be the possible reason for the observed improvement in knowledge and practice scores in the control group. However, this did not significantly change the effect of our intervention on the outcomes, which could be due to the adequate sample size and the low attrition rate [27]. Our intervention had high response rate comparable to other intervention studies among HIV patients in Nigeria [28] and South Africa [29]. In the present study, reasons identified for the retention of respondents were effective communication through calls, text messages and reminder SMS/calls on the days for follow-ups and details of the schedule of subsequent visits provided during the initial contact. These findings are consistent with the report of a study on the retention of participants in clinical trial [30].

### Sensitivity analysis

Sensitivity analysis is vital for considering possible violations of the missing at random assumption. Results of sensitivity analysis of our study with the complete data ignoring cases with missing values and our primary analysis which was based on intention to treat analysis using mean substitution method were similar. The effect sizes in our study was based on partial eta square and Cohen d. The effect sizes in both results were statistically significant in the three outcome variables. This consistency revealed that the results of our findings are robust [17].

### External validity

Several studies have reported strong evidence showing the significant effect of health education intervention in improving knowledge, attitude, and preventive practice regarding tuberculosis in different study population groups from several locations irrespective of socio-demographic differences and cultural diversities [8, 20, 21, and 25]. This is the first study to evaluate the effect of health education intervention in improving knowledge, attitude and practice regarding tuberculosis among people living with HIV in Nigeria. Patients recruited for this study were from one HIV Centre, as such they may not be fully representative of people living HIV in Minna, as due to stigma and discrimination a significant number still access HIV /AIDS services elsewhere. Therefore, the interpretation of the findings of this study should be done with caution because of inherent weaknesses and drawbacks in respect of study design and its implementation. These reasons limit the generalizability of our findings to the HIV population in Nigeria, despite the use of probability sampling and the low attrition rate recorded.

### Strengths

This study was a randomized control trial. The random allocation (RCT) to study groups ensured that every individual that participated in the experiment has an equal chance of been assigned to the experiment/treatment or control groups. This was to ensure beforehand that, as far as possible, the control and treatment groups were similar. Mixed design ANOVA used for this study allowed for measurement of the combination of fixed effects, random effects and repeated measure in the analysis. Intention to treat analysis was used in this study. The effect of health education intervention program has not been evaluated in Nigeria. The significance of this study as a structured health education intervention program on behavior modification that is directed and specific for HIV patients regarding tuberculosis is that it can be considered for adoption in Nigeria.

### Limitations

Despite the effort to control contamination by conducting the training on different days, contamination of the control group could have occurred.

## Conclusion

This study has shown that the health education intervention program is effective in improving knowledge, attitude, and practice regarding tuberculosis among HIV patients. The module developed is recommended to be included as a strategy in the National tuberculosis control guidelines in the training and sensitization of HIV patients on tuberculosis prevention at health facility level.

## Supporting information

S1 File Study protocol. (DOCX)

S2 File Consort checklist. (DOC)
