# Supplementary material for: Effectiveness of health education intervention in improving knowledge, attitude, and practices regarding Tuberculosis among HIV patients in General Hospital Minna, Nigeria – A randomized control trial
Source: PLoS One. 2018 Feb 22;13(2):e0192276. doi: 10.1371/journal.pone.0192276 (PMC5823396; doi:10.1371/journal.pone.0192276)
Supplement: S1 File — (DOCX) [file pone.0192276.s001.docx]

**Effectiveness of health education intervention in improving knowledge, attitude and practices regarding Tuberculosis among HIV patients in General Hospital Minna, Nigeria - A study protocol**

Chindo Ibrahim Bisallah¹, *Lekhraj Rampal¹, Munn-Sann Lye¹, Sherina Mohd Sidik²

¹Department of Community Health, Faculty of Medicine and Health Sciences,

Universiti Putra Malaysia, 43400 Serdang, Selangor, Malaysia

²Department of Psychiatry, Faculty of Medicine and Health Sciences,

Universiti Putra Malaysia, 43400 Serdang, Selangor, Malaysia

* Corresponding Author

Prof. Datuk Dr. Lekhraj Rampal

Department of Community Health, Faculty of Medicine and Health Sciences,

Universiti Putra Malaysia,

43400 Serdang, Selangor, Malaysia

E-mail: [lekhraj@upm.edu.my](mailto:lekhraj@upm.edu.my)

**Abstract**

**Background**

Tuberculosis (TB) is a disease of major public health concern especially in Africa where there is significant upsurge because of the HIV epidemic. It is also the commonest cause of death in AIDS patients. The risk of development of active TB in HIV-infected individuals is 20-37 times higher than those that are HIV negative. Studies have reported that knowledge, attitude and practice (KAP) regarding tuberculosis among HIV patients is poor and has been associated with high transmission. A review of available literature of studies on KAP regarding TB among HIV patients have recommended a health education intervention that is specific and directed at this high risk group. This paper outline the study protocol to evaluate the effectiveness of a new developed health education intervention module in improving knowledge, attitude and practice among HIV patients in Nigeria.

**Methods/design**

A single blind parallel group randomized control trial will be conducted to test and evaluate the effectiveness of a new developed module on KAP regarding TB among HIV patients receiving treatment at General Hospital, Minna, Nigeria. A simple random sampling method will be used in selecting 226 eligible participants. The participants will be randomly allocated to intervention and control group at a ratio of 1:1. The intervention group will receive health education regarding tuberculosis. The control group will received the normal services provided for the HIV patients. Data will be collected using a self-administered questionnaire at baseline, immediate post intervention, three, six and nine months. The data collected will be analyzed using SPSS version 22.The outcome measures will be knowledge, attitude and practice.

**Discussion/ expected implication of the study**

Studies on the effect of health education intervention on KAP regarding tuberculosis among HIV patients are scarce. The effect of health education intervention program has not been evaluated in Nigeria. Advocacy, Communication, and Social Mobilization (ACSM), a component of TB control program in Nigeria was evaluated in 2012 and found to be largely ineffective. This study will test and evaluate the effect of health education intervention program using a theory based approach. If the intervention is effective it will provide a structured behavioral modification intervention program that is directed and specific for HIV patients and will generate valuable information for evaluating and further developing national training program regarding TB that can be adopted nationwide.

**Trial Registration** - [www.pactr.org](http://www.pactr.org) identifier: PACTR201603001403923

**Keyword***s:* Health education intervention, knowledge, attitude, practice, tuberculosis, HIV patients.

Background

Tuberculosis is a disease of major public health concern especially in Africa where there is significant upsurge because of the ravaging HIV epidemic. Tuberculosis is the most common opportunistic infection among HIV infected persons, and remains the most powerful driver of the epidemic. It is also the commonest cause of death in AIDS patients (Olaniran et al 2011).

At the global level people living with HIV are 29 times more likely to develop TB disease than those who are HIV-negative. With the discovery in the 1980s of human immunodeficiency virus (HIV), the HIV epidemic has led to a major upsurge of TB cases and TB mortality in many countries, especially Africa. In 2013, 1.1 million representing 13% of the 9.0 million people who were diagnosed with TB worldwide were HIV-positive and 78% of these HIV-positive TB cases were in the Africa (WHO, 2014).

Nigeria was ranked the 10th in the world and fourth in Africa among the 22 countries with high burden of tuberculosis. The first ever national TB prevalence survey conducted in 2012 revealed a TB prevalence and incidence rates of 322/100,000 and 338/100,000 respectively (National Tuberculosis & Leprosy Control program [NTBLCP], 2012). The global tuberculosis report 2014, WHO estimated the prevalence of TB in Nigeria as 326/100,000 and an incidence rate of 338/100,000 confirming the report of the survey conducted in 2012 (WHO, 2014).

The rising incidence of TB among people living with HIV virus poses a great threat to TB control and indeed a great risk of increase transmission to the general public and the development of multi drug resistant tuberculosis. With HIV prevalence of 3.4% (FMOH, 2012), having a generalized epidemic, Nigeria faces a sustained transmission of tuberculosis until the HIV epidemic is brought under control. One out of every four (4) HIV patients has active tuberculosis in Nigeria with a TB/HIV co-infection rate of 27% with the implication that 35,000 TB cases will be added annually by people living with HIV (FMOH, 2013).

The risk of development of active TB in HIV infected individuals is up to 20-37 times higher than those that are HIV negative. It is estimated that in some countries in sub- Saharan Africa with high HIV prevalence about 80% of TB patients have HIV (Granich et al., 2010, Gyar et al., 2014). The high prevalence of TB among HIV patients from different location in Nigeria of 49%, 10.5%, 16.7% and 34.5% (Jamda et al, 2014, Gyar et al, 2014, Iliyasu & Babashani, 2009, Pennap et al, 2009), poses a great threat to TB control.

Studies have reported that knowledge, attitude and practice (KAP) regarding TB among HIV patients is poor and considered a serious challenge to tuberculosis control program (Gouda et al, 2014, Abebe, 2012). Poor knowledge of TB amongst people living with HIV is associated with high transmission and delay in health seeking behavior (Abebe *et al.* 2012). Ideally HIV patients should have good knowledge regarding TB to prevent infection and development of active disease.

The trend of tuberculosis in Nigeria, based on the graph below shows an upward trend of all forms of TB cases notified from 2002 – 2013. In a nutshell, despite all efforts and current strategies in the control of TB in Nigeria, the fight against TB epidemic is far from being over and is a major source of concern (NTBLCP, 2015).

Source: NTLCP FMOHN (2014).

There is paucity of research to determine TB knowledge and prevention practices among HIV patients in Nigeria. Studies conducted on knowledge, attitude and practice regarding tuberculosis among HIV patients are mainly descriptive, and the studies on effect of health education intervention are scarce.

There is no structured health education intervention program on behavior modification that is directed and specific for HIV patients regarding tuberculosis in Nigeria. Advocacy, Communication and Social Mobilization (ACSM) strategy, a component of TB control program was evaluated in the year 2012 and found to be largely been ineffective (Otu, 2013). Moreover, if nothing is done the dual HIV/TB co-infection will continue to grow, increase death among HIV patients

This study is aimed at evaluating the effectiveness of a new health education intervention program in improving knowledge, attitude and practice regarding tuberculosis among HIV patients in Minna, Niger State, Nigeria based on the principle of information, motivation and behavior theory.

Objectives of study

General objective

To develop, implement and evaluate the effectiveness of health education intervention program in improving knowledge, attitude and practices regarding Tuberculosis amongst HIV patients in General Hospital Minna, Nigeria.

Specific Objectives

1. To determine and compare the socio-demographic (age, gender, educational level, ethnicity, occupation and socio-economic status etc.), psychosocial (anxiety and depression) characteristics and knowledge, attitude and practice of the respondents in the treatment and control groups at baseline.
2. To develop and implement health education intervention program in improving knowledge, attitude and practices regarding tuberculosis among HIV patients in the intervention group.
3. To evaluate the effectiveness of health education intervention program among HIV patients within and between the groups in improving knowledge, attitude and practices regarding tuberculosis at 3, 6 and 9 months follow-up period.

Research Hypothesis

1. There is no significant difference in the socio-demographic characteristics (age, gender, educational level, ethnicity, occupation and socio-economic status etc.), psychosocial factors (anxiety and depression), knowledge, attitude and practice of respondents in the treatment and control groups at baseline
2. Health education intervention program is effective in improving knowledge, attitude and practices regarding tuberculosis among HIV patients within the intervention group compared to the control group at 3, 6 and 9 months follow-up period.

Methodology

Design

A single blind, parallel group, randomized control trial on knowledge, attitude and practices regarding tuberculosis among HIV patients will be conducted in General Hospital, Minna, Nigeria. The trial will evaluate the effect of a new developed module in improving knowledge, attitude and practice regarding tuberculosis among HIV patients. We aim to recruit 226 respondents and randomly allocate them into intervention and control group. The intervention group will receive health education training on tuberculosis, while the control group will receive normal services provided by the ministry of health. Data will be collected at baseline, immediate post intervention, three, six and nine months follow up period. The inclusion criteria for eligibility to participate will be registered HIV patients, including AIDS patients accessing treatment and care at Minna, General Hospital, and age 18 years and above. HIV patients co-infected with tuberculosis or psychiatric disorders or mentally unstable person will be excluded. Ethical approval to conduct the study was obtained from Universiti Putra Malaysia Ethics Committee for Research Involving Human subjects and Niger State Ministry of Health, Human Research Ethics Committee. In addition the trial is registered (Reg. no. PACTR201603001403923) with the Pan African Clinical trial registry located at the South African Medical Research Council (MRC).

Study setting, location and population

The study will be conducted at General Hospital, Minna, Niger state, North-Central, Nigeria. The hospital is the largest referral hospital in the state, established in 1926. It is a 300-bed capacity hospital providing both secondary and tertiary health care services. The hospital provides comprehensive antiretroviral (ART) services including voluntary counseling and testing, prevention of mother to child transmission of HIV, pharmaceutical and laboratory services and capacity building. It also provides a wide range of tuberculosis services including Directly Observed Treatment Short Course Strategy (DOTS), sputum smear microscopy, radiological services, counseling and training. The comprehensive HIV/AIDS treatment centre is the first of its type in the state, established in May 2007, and has registered 8,426 HIV positive patients for treatment and care from 2007 – December, 2014.

Participant recruitment

A simple random sampling method will be used in selecting those that will be eligible to participate in the study. A sample frame will be used which will provide the list of all HIV patients receiving treatment and care in the hospital in the last 2 years. A computer generator program will be used to select a random sample.

Sample size estimation

The sample size of 196 (98 per group) was calculated using the two-proportion formula (Lemeshow et.al, 1990). Knowledge regarding tuberculosis as an outcome variable provided the highest. This was further increased to a final sample size of 226 considering an attrition rate of 15.13% in a similar study (Cornman et al., 2008).

Randomization and blinding

An independent statistician will be given the identification codes of eligible respondents serially numbered. He will subsequently allocate the sampled population into an intervention and a control group using a random number generator program based on simple randomization technique to a ratio of 1:1, each group having 113 participants. Written allocation will be used with identification codes in sealed brown opaque envelopes to ensure allocation concealment. The code numbers will be used to identify participants on the questionnaire while maintaining confidentiality. Informed consent will be obtained after providing details of the study to each potential participant. The participants will not be informed of the random allocation (single blind).

Assessed for eligibility (n=)

Excluded (n=)

1. Not meeting inclusion criteria
2. Declined to participate
3. Other reasons

Randomization and blinding (n=)

Allocated to Control group (n=)

1. Baseline data collection
2. Received allocated intervention(n= )

(Health education on HIV/AIDS)

1. Did not received allocated intervention (n=) & reasons.
2. Immediate post exposure data collection

Allocated to Intervention group (n= )

1. Baseline data collection
2. Received allocated intervention(n= )

(Health education on tuberculosis)

1. Did not received allocated intervention (n=) & reasons.
2. Immediate post exposure data collection

3 months follow up (n=)

1. Loss to follow up or discontinued intervention (n= -reasons)
2. Data collection

3 months follow up (n=)

1. Loss to follow up(n= ) or discontinued intervention (n= ) - reasons
2. Data collection
3. Repeat of intervention

6 months follow up (n=)

1. Data collection

6 months follow up (n=)

1. Data collection

9 months follow up (n =)

1. Data collection

9 months follow up (n=)

1. Data collection

ANALYZED (n=)

ANALYZED (n=)

**Fig.1 Study flow chart.**

Intervention module

The health education intervention module on knowledge, attitude, and practice regarding tuberculosis among HIV patients was developed through a process of consultations with a group of experts in preventive medicine and behavior modification. The module was developed based on the Information Motivation Behavior (IMB) model which has three components as determinants of health behavior change including information, motivation and behavioral skills (Fisher et al, 2009). The information component of the module provided information on basic facts about tuberculosis, modes of transmission, prevention, risk factors, vulnerability and misconception related to TB. The motivation component of the model was aimed at countering and refuting misconceptions about tuberculosis leading to positive attitudinal change. The preceding steps will motivate individuals to access screening and preventive services provided free of charge to HIV patients. Knowledge acquired during the training is expected to reinforce preventive behavior skills.

Intervention

A structured health education intervention program, aimed at introducing a systematic way of educating HIV patients to prevent infection and development of tuberculosis will be conducted for the intervention group. The control group will receive the normal services provided by the Ministry of Health, Positive health, dignity and prevention of HV/AIDS. An agenda will be developed for the training for each group. The program will essentially be a teaching exercise using PowerPoint presentation, brainstorming, questions and answer session. The program will be delivered in two days, one day each for the control and intervention group. Six Facilitators from the TB control program, agency for the control of AIDS and General Hospital, Minna will be trained to deliver the module. Facilitators training will be conducted two weeks before the exercise. The participants in the two groups will be divided into a class of not more than 38 participants for ease of delivery and interraction. The intervention will be given at the first encounter and a booster at 3 months to reinforce learning. Questionnaire for data collection will be administered at baseline (pre and post), three months, six months and nine months follow up. The training will be conducted in the hospital premises and participants will be provided meals and transport allowance.

Assessment of respondents

To determine knowledge, attitude and practice, the questionnaire will be adapted and modified from the Validated WHO/Stop TB partnership Advocacy, Communication and Social Mobilization for Tuberculosis control: A guide to developing Knowledge, Attitude and Practice surveys(WHO, 2008). The second part of the questionnaire on anxiety and depression will be adapted from the validated Hospital and anxiety and depression (HAD) rating scale among HIV/AIDS patients at Kano, Nigeria (Sale et al, 2014). The questionnaires will be self-administered and data will be collected from respondents at baseline, immediate post intervention, three, six and nine months' post-intervention. Those that cannot read or write will be assisted and questions read out to them in the local language (Hausa) as translated and filled according to their choice.   The questionnaire will have six section, A, B, C, D, E and F covering socio-demographic variables, TB-related knowledge, attitude towards TB, practices related to TB, anxiety and depression, clinical and laboratory parameters respectively. Section A will have 13 statements on socio-demographic variables. The section on TB knowledge will have 24 statements with ‘Yes' or 'No' options. Section C will measure respondents attitude towards tuberculosis on a 5-point Likert scale with the options: strongly agree, agree, neutral, disagree and strongly disagree. Section D will assess respondents practices of patients relating to tuberculosis prevention with 'Yes' or 'No' options. Section E will have statements on the hospital anxiety and depression (HAD) scale with the options 0,1,2,3 for each statement. The last section E will contained information relating to the patient clinical and laboratory parameters. The researcher and the facilitators will conduct the follow up assessments using same questionnaire for the two arms of the study.

Data analysis

Data will be analyzed using Statistical package for social sciences (SPSS) version 22. Exploratory data analysis will be conducted in SPSS to clean the data and ensure that errors did not occur during data entry, identify outliers and missing data. Normality tests will be conducted for all continuous variables. Parametric test such as Independent t-test, mixed design ANOVA and Nonparametric test (Chi-square, Fisher exact test and McNemar test) will be used to analysis the data. The p-values of the results of the analysis will be compared to an alpha (α) level of significance of 0.05 (95% confidence interval) and the power of 80% to detect the impact of intervention. Chi-square and Fisher exact test will be used to analyze categorical variables. Mixed design ANOVA will be applied as the major analytical method to determine the effectiveness of the intervention.

Expected Outcomes

The expected outcomes of the intervention program will be a significant improvement in knowledge, attitude, and practices regarding TB among the respondents in the intervention group as compared to the control group. A manual for educating of HIV patients regarding TB produced.

**Discussion**

The main aim of this study is to evaluate the effectiveness of health education intervention program in improving knowledge, attiyude and practice regarding tuberculosis among HIV patients. Review of previous literature has shown that knowledge, attitude and preventative practice regarding TB among this high-risk group is poor and considered a serious threat to TB prevention and control program. These studies have also recommended a specific and targeted education intervention program to this group. Review of advocacy, communication, and social mobilization strategy, a component of National TB control program that provide information and education to the general public regarding TB was found to be largely ineffective. Hence the need to test the effect of a new intervention targeting this highly vulnerable group.

The strengths of the study include the randomized control trial design and the long term follow up starting from baseline, immediate post intervention assessment, and three, six and nine months follow up assessment.

The findings of this study will contribute to the body of knowledge and will generate valuable information for evaluating and further developing national training programs regarding TB. It will benefit HIV patients by heightening their level of awareness about TB, which will influence attitudinal change and reduce the risk of infection or development of active disease. It is expected to influence policy decision by government and international development partners for the inclusion of new strategy for the prevention and control of TB among HIV patients which presently does not exist. This intervention will serve to bridge the gap between information and accessibility of these patients to the existing TB services

References

. Abebe R, Demissie M. Assessment of knowledge and practices related to tuberculosis and associated factors among HIV positive people in Addis Ababa, Ethiopia. Global Journal of Medicine and Public Health 2012; 1(2):35-42

Cornman DH, Kiene SM, Christie S, Fisher WA, Shuper PA, Pillay S, Friedland GH, Thomas CM, Lodge L, Fisher JD. Clinic-based intervention reduces unprotected sexual behavior among HIV-infected patients in KwaZulu-Natal, South Africa: results of a pilot study. Journal of acquired immune deficiency syndromes 2008; 48(5):553.

Fisher .J.D., Laramie S. (2009). Secondary Prevention of HIV Infection: the current State of prevention for positives. Curr Opin HIV and AIDS. 4(4): 279–287.

FMOH. (2013). Nigeria Stop TB Partnership Strategic plan 2013-2015.

Gouda, S., Peerapur, B., Rudramma, J., Kaleem, a, & Sandhya, R. (2014). A study to determine and compare the knowledge, attitude and compliance of Tuberculosis treatment among HIV seropositive and HIV seronegative TB patients. BMC Infectious Diseases, 14(3), 9.

Granich, R., Akolo, C., Gunneberg, C., Getahun, H., Williams, P., & Williams, B. (2010). Prevention of tuberculosis in people living with HIV. Clinical Infectious Diseases 50( 3) 215–S222.

Gyar, S. D., Dauda, E., & Reuben, C. R. (2014). Prevalence of Tuberculosis in HIV/AIDS Patients in Lafia, Central Nigeria. Int. J. Curr. Microbiol. App. Sci, 3(6), 831-838.

Iliyasu, Z., & Babashani, M. (2009). Prevalence and predictors of tuberculosis coinfection among HIV-seropositive patients attending the Aminu Kano Teaching Hospital, northern Nigeria. Journal of epidemiology, 19(2), 81-87

Lemeshow, S., David. W. H., Klar, J., & Lwanga, S. K. (1990). Adequacy of sample size in health studies. John wiley & sons Ltd.

NTBLCP. (2012). Report First National TB Prevalence Survey 2012, Nigeria.

NTBLCP. (2015). The national strategic plan for tuberculosis control 2015-2020, towards universal access to prevention, diagnosis and traeatment.

Olaniran, O., Oyovwevotu, M. A, & Agunlejika, R. A. (2011). Prevalence of Tuberculosis among HIV / AIDS patients In Obafemi Awolowo University Teaching Hospital Complex Oauthc , Ile – Ife. International Journal of Biological & Medical Research, 2(4), 874–877.

Otu, A. (2013). A review of the national tuberculosis and leprosy control programme of Nigeria: Challenges and prospects. Annals of Tropical Medicine and Public Health. 6(5):491.

Pennap, G. R., Giyan, J. N., & Eleboda, A. T. (2009). Prevalence of pulmonary tuberculosis among people living with HIV/AIDS in Keffi and its environs. Indian Journal of Microbiology, 49, 233–236.

Sale S, Dankishiya F. S, Gadanya, M.A (2014) "Validation of hospital Anxiety and depression rating scale among HIV/AIDS patients in Aminu Kano Teaching Hospital, Kano, North Western Nigeria." Journal of Therapy and Management in HIV Infection, 2, 45 - 49.

WHO. (2008). A guide to developing knowledge , attitude and practice surveys. World Health Organisation.

WHO. (2014). Global tuberculosis report 2014 (WHO/HTM/TB/2014.08). doi:WHO/HTM/TB/2014.08

/
